# Involvement of Essential Signaling Cascades and Analysis of Gene Networks in Diabesity

**DOI:** 10.3390/genes11111256

**Published:** 2020-10-25

**Authors:** Udhaya Kumar S., Bithia Rajan, Thirumal Kumar D., Anu Preethi V., Taghreed Abunada, Salma Younes, Sarah Okashah, Selvarajan Ethiraj, George Priya Doss C., Hatem Zayed

**Affiliations:** 1 School of BioSciences and Technology, Vellore Institute of Technology, Vellore 632014, Tamil Nadu, India; s.udhayakumar2018@vitstudent.ac.in (U.K.S.); bithia0205@gmail.com (B.R.); thirumalkumar.d@gmail.com (T.K.D.); 2 School of Computer Science and Engineering, Vellore Institute of Technology, Vellore 632014, Tamil Nadu, India; anupreethi.v2019@vitstudent.ac.in; 3 Department of Biomedical Sciences, College of Health and Sciences, QU Health, Qatar University, Doha 2713, Qatar; taghreed.abunada@qu.edu.qa (T.A.); sy1203986@student.qu.edu.qa (S.Y.); so1404563@student.qu.edu.qa (S.O.); 4 Department of Genetic Engineering, Kattankulathur Campus, SRM Institute of Science and Technology, Chennai 603203, Tamil Nadu, India; selvarajan.e@ktr.srmuniv.ac.in

**Keywords:** diabetes, T2D, T1D, MODY, obesity, diabesity, protein-protein interaction

## Abstract

(1) Aims: Diabesity, defined as diabetes occurring in the context of obesity, is a serious health problem that is associated with an increased risk of premature heart attack, stroke, and death. To date, a key challenge has been to understand the molecular pathways that play significant roles in diabesity. In this study, we aimed to investigate the genetic links between diabetes and obesity in diabetic individuals and highlight the role(s) of shared genes in individuals with diabesity. (2) Methods: The interactions between the genes were analyzed using the Search Tool for the Retrieval of Interacting Genes (STRING) tool after the compilation of obesity genes associated with type 1 diabetes (T1D), type 2 diabetes (T2D), and maturity-onset diabetes of the young (MODY). Cytoscape plugins were utilized for enrichment analysis. (3) Results: We identified 546 obesity genes that are associated with T1D, T2D, and MODY. The network backbone of the identified genes comprised 514 nodes and 4126 edges with an estimated clustering coefficient of 0.242. The Molecular Complex Detection (MCODE) generated three clusters with a score of 33.61, 16.788, and 6.783, each. The highest-scoring nodes of the clusters were *AGT*, *FGB*, and *LDLR* genes. The genes from cluster 1 were enriched in FOXO-mediated transcription of oxidative stress, renin secretion, and regulation of lipolysis in adipocytes. The cluster 2 genes enriched in Src homology 2 domain-containing (SHC)-related events triggered by *IGF1R*, regulation of lipolysis in adipocytes, and GRB2: SOS produce a link to mitogen-activated protein kinase (MAPK) signaling for integrins. The cluster 3 genes ere enriched in IGF1R signaling cascade and insulin signaling pathway. (4) Conclusion: This study presents a platform to discover potential targets for diabesity treatment and helps in understanding the molecular mechanism.

## 1. Introduction

Diabesity is a term that describes a causal pathophysiological link between obesity and diabetes [1]. In the last two decades, there has been a substantial rise in the number of people diagnosed with obesity and diabetes. This pattern forced a tremendous burden on medical care systems and is expected to continue growing during the next decades [2]. Diabesity is currently considered the leading cause of modern chronic diseases, such as cardiovascular diseases, strokes, dementia, and cancer. The concept of diabesity has been linked to impaired pathways of metabolic cell signaling as well as altered insulin signaling, i.e., insulin resistance (IR), which upsurges the risk of developing type 2 diabetes (T2D) [3,4]. People with obesity, T2D, and diabesity have high levels of circulating extracellular vesicles (EVs). Various metabolic pathways, including the Phosphoinositide 3-kinases (PI3K)/ Protein Kinase B (Akt) pathway, MAPK, and mammalian target of rapamycin (mTOR) signaling, are altered by the influence of EVs on cellular and systemic responses [4]. In addition, diabesity-linked brain alterations are a consequence of disorders in insulin signaling and mitochondria, which upsurges the risk of developing neurodegenerative diseases [5]. Further, diabesity is strongly linked with an increased risk of primary human cancers [5,6,7]. Moreover, diabesity has been shown to induce inflammation and increase adenosine concentration, which, in turn, increases cell proliferation [7]. Individuals at risk of developing diabesity demonstrate a family history of T2D, earlier maternal record of diabetes, gestational diabetes mellitus (GDM), being small-for-gestational age at birth. They might also display signs of insulin resistance or other related conditions, including hypertension, dyslipidemia, polycystic ovary syndrome (PCOS). Patients with obesity, T2D, or diabesity have been shown to demonstrate endoplasmic reticulum stress, with noticeable effects on visceral adipose tissue (VAT) adipose tissue, fetoplacental vascular endothelium, liver, and skeletal muscles [8]. These complications of diabesity have emerged as significant threats in developing and under-developed nations, particularly driven by the worldwide rise in obesity rates.

Given the fact that obesity and T2D are closely associated, identifying the genetic link between these two complex polygenic diseases may help understand the driving factors of diabesity. Recently, three hypotheses have been developed to explain the molecular mechanisms of diabesity [9]: (i) The “inflammation hypothesis” states that obesity signifies a chronic inflammatory condition in which the inflammatory molecules produced by infiltrating macrophages in adipose tissue trigger pathological variations in insulin-sensitive tissues and β-cells [10,11]. (ii) The “lipid overflow hypothesis” states that obesity may result in upsurged ectopic lipid storage because of the restricted capacity of adipose tissue to appropriately store fat in obese individuals. Potentially damaging lipid components and metabolites may apply cytotoxic impacts on peripheral cells [12,13] (iii) The “adipokine hypothesis” states that the main trait of white adipose cells is to serve as an endocrine organ and to release variable hormones with auto- and paracrine-function. Increasing fat storage can trigger the dysfunctional release of endocrine factors, thereby causing metabolic damage of insulin target tissues and, ultimately, insulin yielding β-cells failure [14].

In 2010, the global estimate of people with diabetes was between 150-220 million. By 2040, it is projected to increase to 642 million [15]. The prevalence of T2D has increased dramatically during the last two decades, driven by the global increase in the number of obese individuals. Obesity and T2D are underpinned by a combination of genetic and environmental factors. Unhealthy eating habits and lifestyles are major environmental factors that contribute to T2D development [16]. Obesity is a significant contributor to lipid and glucose metabolic dysfunction, and it is known to cause organ dysfunction on a wider scale, affecting cardiac, liver, pulmonary, endocrine, and reproductive functions. Because of the consequences of the secretion of inflammatory adipokines, obesity is associated with immune dysfunction and is a major contributor to several types of cancer [17]. The pathophysiology of T2D includes microvascular complications (diabetic nephropathy, retinopathy, and neuropathy) as well as macrovascular complications (cerebrovascular disease, atherosclerosis, and cardiovascular disease) [18]. Microvascular complications involve abnormal glycemia, while macrovascular complications are unrelated to abnormalities in glycemia; however, hyperglycemia increases the risk of developing macrovascular complications [19]. It is extremely important to understand the role of genetic variations to be able to assess the susceptibility of an individual to identified risk factors. Genetic variations may describe why T2D affects only a small fraction of obese people when the majority of people with diabetes are obese. Candidate obesity genes and traits associated with obesity include *LEP, MCR4*, *POMC*, and *PCSK1* [20,21]. Multiple candidate obesity genes, such as *FTO*, *NPC1*, *MC4R*, *SH2B1*, *NRX3*, *POMC*, *NEGR1,* and *BDNF,* were shown to be involved in adipose tissue development, function, and hyperphagia regulation [22]. Obesity-related genes impact hormones and associated peptide production, including LEP, IGF2, and IGF1, and receptors, such as IGF1R, AR, FSHR, ESR1, and LEPR [21]. The genes associated with T2D include *PPARG*, *KCNJ11*, *TCF7L2*, *FTO*, *IGF2BP2*, *KCNQ1*, *NOTCH2*, *HNF1A*, and *HNF1B* [23]. *ABCC8* and *KCNJ11* control insulin secretion through the ATP sensitive potassium channel, polymorphisms in these genes are associated with diabetes [24,25,26]. T2D is believed to have a different genetic architecture in obese and non-obese individuals [27]. Maturity-onset diabetes of the young (MODY), a monogenic form of diabetes, can result from mutations in one of the genes expressed in β-cell that include *GCK* [28], *HNF1A* [29], *HNF1B* [30], *HNF4A* [31], *PDX1* [32], and *B2M* [33]. These genes contribute to the transcription regulation of enzyme-encoding genes involving the transport and metabolism of glucose and other proteins essential for the normal function of β-cells [34]. The genes associated with MODY may contribute to the polygenic nature and development of T2D. A key genetic factor associated with T2D may be variants in the MODY-associated gene, *HNF1A* [35]. *FTO* is one of the best examples of a diabetogenic gene that promotes its impact through obesity. *FTO* was first recorded as an obesity susceptibility gene and was later reported as a T2D associated body mass index (BMI) gene [36]. Another linking mechanisms of obesity and diabetes involve the T-bet transcription factor [37], as well as via mitochondrial dysfunction [38,39]. The molecular pathways underlying the two complex polygenic disorders are still far from being well understood, considering the identification of several candidate genes for both obesity and diabetes. A comprehensive understanding of the underlying genetic influence and protein-protein networks will enable us to understand the molecular etiology of diabesity and thereby help manage and eventually prevent or cure diabesity.

## 2. Materials and Methods

### 2.1. Data Source

The dataset for the current research was gathered from obesity and co-morbid diseases database (OCDD) (http://www.isical.ac.in/~systemsbiology/OCDD/home.php) [40], PubMed, and Google Scholar. The compilation of obesity genes associated with Type-1 Diabetes (T1D), T2D, and maturity-onset diabetes of the young (MODY) was carried out using keywords “Obesity” AND “Type 1 Diabetes”, “Obesity” AND “Type 2 Diabetes” and “Obesity” AND “MODY” within the three data sources. These database search provided us with various obesity genes linked with T1D, T2D, and MODY. All genes were collected and manually curated for further process.

### 2.2. Circos Plot Construction and Data Visualization

Circos, a visualization software for comparative genomics [41], was used to identify overlapping genes. The Biomart package of R language was used to obtain the chromosomal information of the genes. To obtain the required cytoband for the circular representation of the data, genome University of California Santa Cruz (UCSC’s) table browser was used (http://genome.ucsc.edu/cgi-bin/hgTables) [42]. An online software, “shinyCircos”, was used to get the circular representation of the given data (http://shinycircos.ncpgr.cn/) [41]. A Venn diagram for all the genes belonging to T1D, T2D, and MODY, was plotted using an online server (http://bioinformatics.psb.ugent.be/webtools/Venn/).

### 2.3. PPI Network Construction and Visualization

The protein-protein interactions (PPI) network of the obesity genes associated with T1D, T2D, and MODY were constructed with the help of the online Search Tool for the Retrieval of Interacting Genes (STRING) database [43]. The STRING database provides a thorough assessment and incorporation of both physical (direct) and functional (indirect) PPIs. As STRING supports queries for multiple proteins, a list of the genes linking obesity and diabetes (T1D, T2D, and MODY) were uploaded to the STRING database, and the search was restricted to *Homo sapiens*. The functional interaction between the query proteins was obtained using a high level of confidence (score ≥ 0.90). The interactions between the query proteins were obtained from STRING as a tab separated value (*tsv*) file. Cytoscape was used for the visualization of the PPI by importing the *tsv* file of the STRING database [44]. Cytoscape supports a range of automated algorithms for the network layout and helps to connect the query network to databases for functional annotations. Cytoscape helps to organize the imported network as a graph by representing the molecular species in the form of nodes and edges, where each node represented a protein product of single-gene and edges represented the protein-protein association. The interactions with excellent data support (high confidence ≥ 0.9) were selected for precision after hiding the disconnected nodes for PPI network construction. This network was then exported for visualization in Cytoscape. The GeneMANIA force-directed layout of the Cytoscape application was used for the display of the PPI network.

### 2.4. Identification of Protein Complexes and Pathways

The Molecular Complex Detection (MCODE) plugin of the Cytoscape app was used to identify the densely connected regions/clusters in the PPI network [45]. MCODE, a clustering algorithm, uses a vertex-weighted graph to identify the molecular complexes from the given PPI network as a whole. With advanced options set as default, the complexes generated from the algorithm were scored, ranked, and further processed to create a cluster network. The top three gene clusters of the interactive network were extracted according to their scores. ClueGO [46] and Cluepedia [47], Cytoscape plugins, facilitated the biological interpretation and visualization of functionally grouped terms of the selected gene clusters in the form of networks. ClueGO incorporates Gene Ontology (GO) terms and Kyoto Encyclopedia of Genes and Genomes (KEGG) pathways to generate a functionally structured GO/pathway term network from the selected clusters by using kappa score ≥ 0.4. The analytical parameters used for annotation network generation were used as predefined by ClueGO. In conjunction with ClueGO, Cluepedia offers a detailed overview of the biological pathways or molecular mechanisms of the selected clusters based on GO, KEGG, and Reactome. With different levels of specificity criteria allowed for the visualization of the GO/pathway term network, we used a cutoff *p*-value ≤ 0.05 for visualizing the different pathways.

## 3. Results

### 3.1. Genes Associated with Diabesity

**Figure 1:**
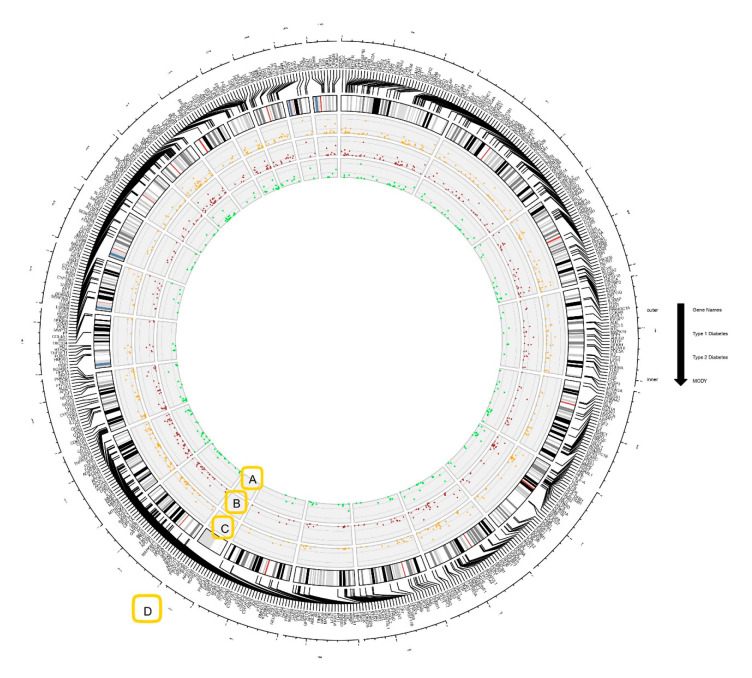
Circos plot demonstrating the genes associated with type-1 diabetes (T1D), type-2 diabetes (T2D), and maturity-onset diabetes of the young (MODY). The percentage of connectivity of genes in (**A**) MODY (green scatter points), (**B**) T2D (red scatter points), and (**C**) T1D (orange scatter points). (**D**) The group of genes from the same chromosome number are represented outside the Circos. The chromosome representation was provided by ideograms from a cytoband file format and imported to shinyCircos.

**Figure 2:**
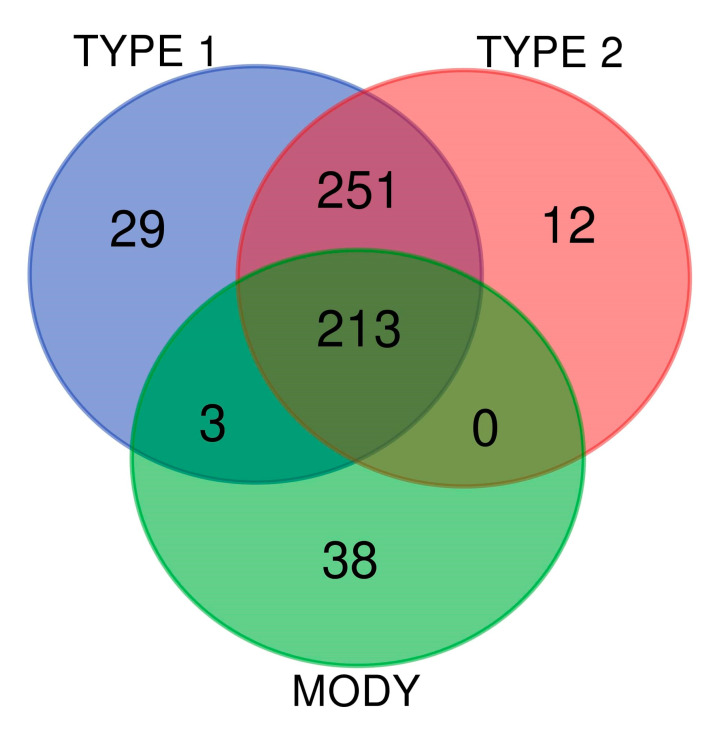
The Venn diagram of gene intersection between the three gene sets of T1D, T2D, and MODY. The plot depicts the similarities and differences between the obesity-associated genes among T1D, T2D, and MODY. The blue, red, green circles represent T1D, T2D, and MODY, respectively.

The OCDD database, along with other literature databases, PubMed, and Google Scholar, assisted our study by providing a comprehensive list of genes linking obesity and diabetes. Manual curation was performed for collected genes, and compilation of obesity genes associated with T1D, T2D, and MODY yielded 546 genes. For further processing, we used these 546 genes for gene networking and functional enrichment analysis. The Circos plot construction and data visualization showed that out of the 546 genes, a total number of 496 genes were associated with T1D, with connectivity rates ranged from 0.01% to 1.59% (Figure 1). In the case of T2D, 476 genes were mapped with connectivity ranged from 0.01% to 1.60% (Figure 1). In MODY, 254 genes whose percentage of connectivity ranged from 0.03% to 2.61% were found (Figure 1). The *INS* gene demonstrated the highest percentage of interconnectedness in the three gene networks for T1D (1.59%), T2D (1.60%), and MODY (2.61%). In this analysis, all the identified obesity genes associated with T1D, T2D, and MODY were interspersed across the human chromosome. We plotted a Venn diagram to illustrate the associations between the identified obesity genes. The results exhibited 213 genes that were shared between T1D, T2D, and MODY (Figure 2 and Supplementary Table S1). This plot uses circles to display the associations between the genes described, and overlapped circles have shared genes between the different phenotypes.

### 3.2. PPI Network Construction and Visualization

**Figure 3:**
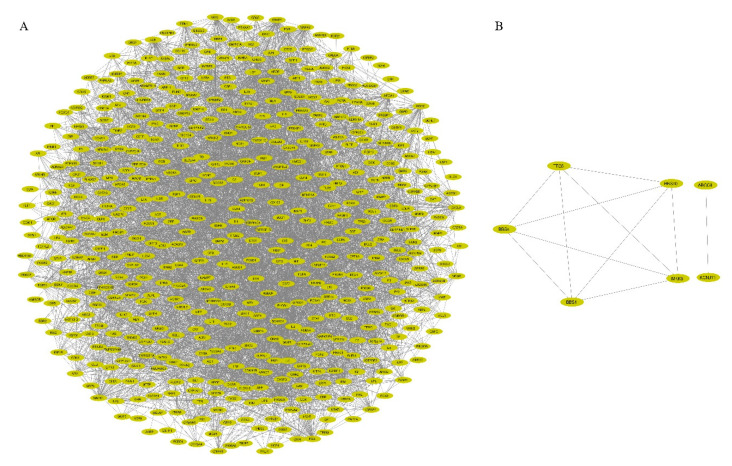
The protein–protein-interacting networks of genes associated with diabesity. (**A**) The network backbone of the identified genes comprised 514 nodes and 4126 edges with an estimated clustering coefficient of 0.242 using Cytoscape. (**B**) The two disconnected networks with 5 and 2 nodes, respectively. Ellipse represents the nodes, and the edges are shown as lines.

The STRING database derived the interactions between the query gene products from known experimental and in silico methodologies. The PPI network was represented in the form of nodes and edges, where each node represented a protein product of single-gene and edges represented the protein-protein association. The interactions with excellent data support (high confidence ≥ 0.9) were selected for precision after hiding the disconnected nodes for PPI network construction. The gene/protein interaction network consisted of 514 nodes (proteins) and 4126 edges (interactions) with an estimated clustering coefficient of 0.242 (Figure 3A,B). As a result, the protein network provided a combined score (≥0.9) for each gene and exhibited the interacting partners. Further, the protein network was analyzed with the NetworkAnalyzer plugin from Cytoscape to elucidate the topological parameters of the identified genes (Table 1). The simple parameters were built from the protein network resulting in clustering coefficient, node numbers, characteristic path length, network heterogeneity, centralization, density, and diameter. These results further helped us to delineate the complex network with the help of the MCODE plugin.

### 3.3. Identification of Protein Complexes and Pathways

**Figure 4:**
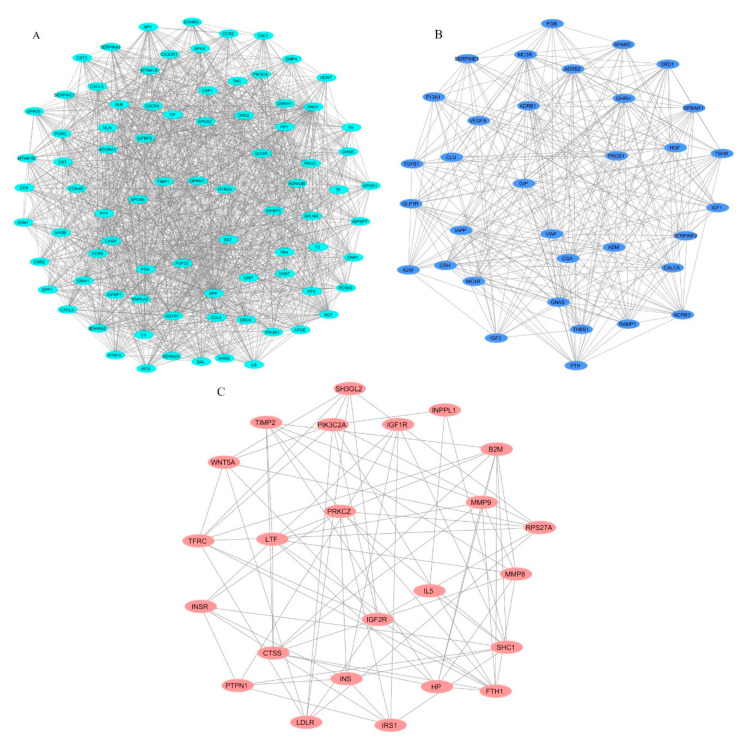
The top three clusters derived from the protein-protein interactions (PPI) network using MCODE. (A) Cluster 1 (score = 33.61), (B) Cluster 2 (score = 16.788), and (C) Cluster 3 (score = 6.783). Ellipse and lines represent the nodes and edges, respectively. Here, the clusters indicate direct PPIs in genes by multiple partners.

For a detailed analysis of the extensive PPI network, the extraction of dense regions around a protein from the PPI network identifies functionally related protein groups. The top three MCODE-generated clusters scored 33.61, 16.788, and 6.783, respectively, based on the vertex weighting of the MCODE algorithm. The first cluster scoring 33.61, was calculated from the seed *AGT* with 83 nodes and 1378 edges. The second cluster scoring 16.788 was derived from *FGB* with 34 nodes and 277 edges. The third cluster scoring 6.783 was derived from *LDLR* with 24 nodes and 78 edges. The intra-cluster networks derived from these seeds are represented (Figure 4A–C). The identified clusters, their scores, and respective node IDs are described (Table 2).

**Figure 5:**
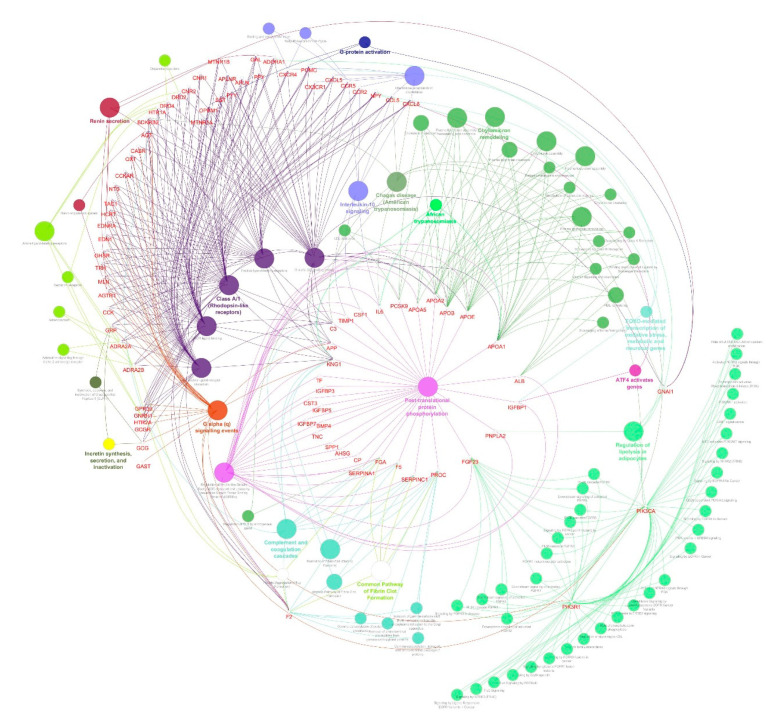
Molecular enrichment networks of genes associated with diabesity and the cluster 1 network enrichment analysis. Visualization of GO gene enrichment profiling using Cytoscape on the basis of ClueGO/CluePedia network processing deciphered from cluster 1. A combination cluster enrichment analysis, such as the GO BF, MF, and KEGG pathway, was provided by the plugin. GO term network connectivity identified by gene-shared edges and cohesive clusters (kappa score ≥ 0.4) and showing pathways (*p*-value ≤ 0.05). The node size indicates the *p*-value. The color code of nodes corresponds to the functional group to which they belong. Bold colored characters signify the most essential functional terms which define the pathways within each class. Each node constitutes a precise term for cluster 1.

**Figure 6:**
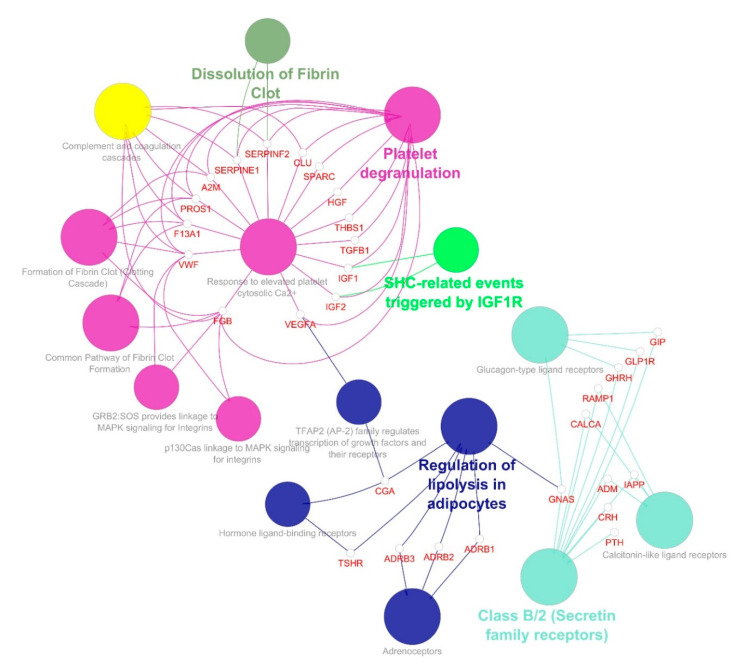
Molecular enrichment networks of genes associated with diabesity and the cluster 2 network enrichment analysis. Visualization of GO gene enrichment profiling using Cytoscape on the basis of ClueGO/CluePedia network processing deciphered from cluster 2. A combination cluster enrichment analysis, such as the GO BF, MF, and KEGG pathway, was provided by the plugin. GO term network connectivity identified by gene-shared edges and cohesive clusters (kappa score ≥ 0.4) and showing pathways (*p*-value ≤ 0.05). The node size indicates the *p*-value. The color code of nodes corresponds to the functional group to which they belong. Bold colored characters signify the most essential functional terms which define the pathways within each class. Each node constitutes a precise term for cluster 2.

**Figure 7:**
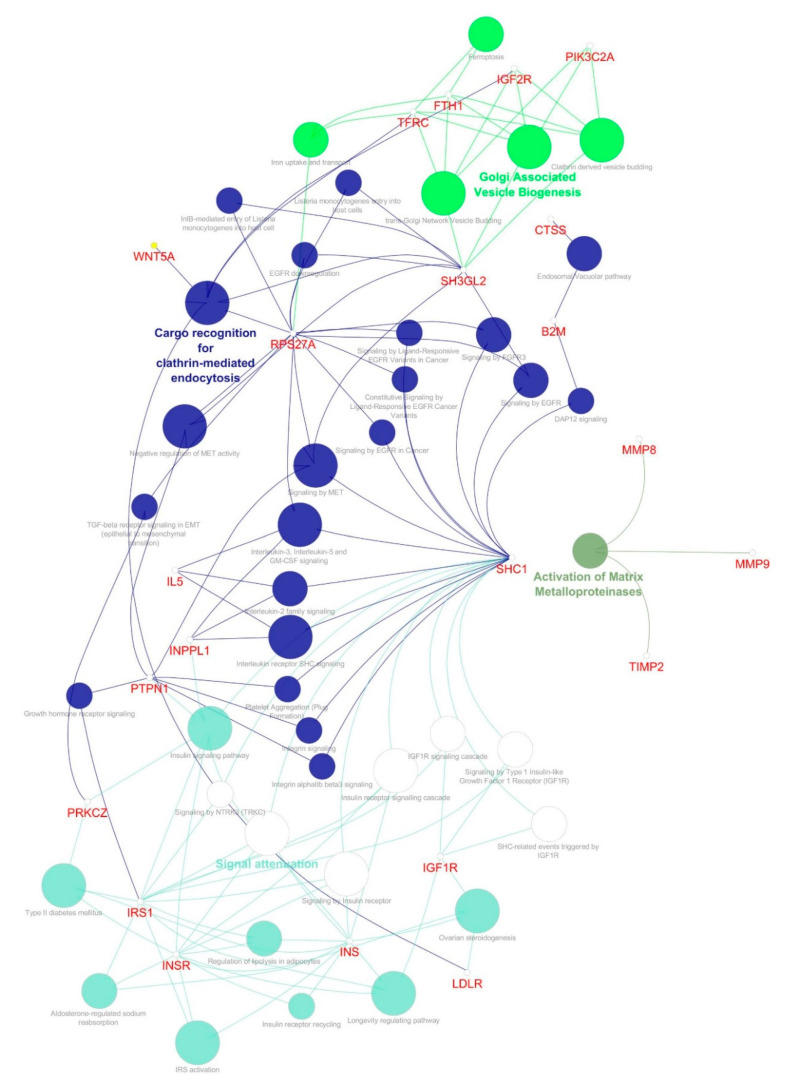
Molecular enrichment networks of genes associated with diabesity and the cluster 3 network enrichment analysis. Visualization of GO gene enrichment profiling using Cytoscape on the basis of ClueGO/CluePedia network processing deciphered from cluster 3. A combination cluster enrichment analysis, such as the GO BF, MF, and KEGG pathway, was provided by the plugin. GO term network connectivity identified by gene-shared edges and cohesive clusters (kappa score ≥ 0.4) and showing pathways (*p*-value ≤ 0.05). The node size indicates the *p*-value. The color code of nodes corresponds to the functional group to which they belong. Bold colored characters signify the most essential functional terms which define the pathways within each class. Each node constitutes a precise term for cluster 3.

To determine the GO terms and enriched pathways from the identified genes, we annotated all three clusters using ClueGO. The ontologies are annotated based on the inbuilt function from ClueGO, which resulted in cellular components, molecular function, biological process, KEGG, and REACTOME categories. The enrichment and pathway analysis showed that the genes from cluster 1 were enriched in FOXO-mediated oxidative stress transcription, uptake by insulin-like growth factor binding proteins (IGFBPs), and regulation of insulin-like growth factor (IGF) transport, renin secretion, Interleukin-10 signaling, transport of γ-carboxylated protein precursors from the endoplasmic reticulum to the Golgi apparatus, platelet aggregation, cholesterol metabolism, plasma lipoprotein assembly–remodeling–clearance, chylomicron assembly–remodeling–clearance, metabolism of fat-soluble vitamins, low-density lipoprotein (LDL) clearance, high-density lipoprotein (HDL) remodeling, regulation of lipolysis in adipocytes, complement and coagulation cascades and others (Figure 5). The cluster 2 genes enriched in SHC-related events triggered by IGF1R, regulation of lipolysis in adipocytes, a common pathway of fibrin clot formation, and GRB:SOS produce a link to MAPK signaling for integrins (Figure 6). The cluster 3 genes enriched in the insulin signaling pathway, IGF1R signaling cascade, regulation of lipolysis in adipocytes, insulin receptor signaling cascade, integrin signaling, Interleukin-2,3,5, signaling, Interleukin receptor SHC signaling, cargo recognition for clathrin-mediated endocytosis and growth hormone receptor signaling (Figure 7).

Cluster 1 seed demonstrated that *AGT* is involved in the renin secretion and renin-angiotensin system. Around 32 genes from cluster 1, including *APOA1, APOA2, APOB, APOE, FGA, IGFBP1*, and, *IL6* are involved in uptake by Insulin-like Growth Factor Binding Proteins (IGFBPs) and the regulation of Insulin-like Growth Factor (IGF) transport. The apolipoproteins of cluster 1 *APOB, APOA1, APOA2, APOE, APOA5,* and *PCSK9* are involved in cholesterol metabolism, plasma lipoprotein assembly–remodeling–clearance, chylomicron assembly–remodeling–clearance, metabolism of fat-soluble vitamins, LDL clearance, HDL remodeling. The regulation of lipolysis in adipocytes involves six genes: *ADORA1, GNAI1, NPY, PIK3CA, PIK3R1,* and *PNPLA2*. Cluster 2 seed, *FGB* is involved in platelet degranulation and response to elevated platelet cytosolic Ca2+, along with *A2M, CLU, F13A1, HGF, IGF1, IGF2, PROS1, SERPINE1, SERPINF2, SPARC, TGFB1*, *THBS1, VEGFA,* and *VWF* genes. *IGF1* and *IGF2* are involved in SHC-related events triggered by *IGF1R* genes. Regulation of lipolysis in adipocytes involved *ADRB1, ADRB2, ADRB3, CGA, GNAS,* and *TSHR*. *INS, INSR,* and *IRS1* of cluster 3. The insulin signaling pathway of cluster 3 involved *INPPL1, INS, INSR, IRS1, PRKCZ, PTPN1,* and *SHC1*. *INS, INSR, IRS1*, and *SHC1* were involved in the insulin receptor signaling cascade. *PTPN1* and *SHC1* were involved in platelet aggregation/plug formation. Supplementary Tables S2–S4 show the detailed enriched pathways along with the corrected *p*-value (Bonferroni step down),% of associated genes, and the number of genes for clusters 1, 2, and 3, respectively. Each signaling cascade involves essential genes and influences other molecular pathways through protein-protein interaction. However, in concern with diabesity interlinked signaling cascades, it is highly suggestible to focus and characterize each dysregulated pathway that might implicate directly/indirectly to obesity-associated diseases, such as T1D, T2D, and MODY.

## 4. Discussion

Several studies have revealed a considerable number of diabesity-associated susceptibility genes; however, the potential mechanisms underlying this complex disorder remain poorly understood. The proteins encoded by susceptibility genes may determine an individual’s susceptibility to diabesity via their encoded PPIs. In this study, a total of 546 genes that are associated with diabesity were identified, and a PPI network using STRING was generated (Figure 3). We identified a network of genes that comprised 514 nodes and 4126 edges with an estimated clustering coefficient of 0.242. Based on the vertex weighting of the MCODE algorithm, we identified three clusters with scores of 33.61, 16.788, and 6.783, each. Genes from cluster 1 were enriched in FOXO-mediated transcription of oxidative stress, renin secretion, and regulation of lipolysis in adipocytes. Cluster 2 genes enriched in SHC-related events triggered by IGF1R, regulation of lipolysis in adipocytes, and GRB2: SOS produce a link to MAPK signaling for integrins. Cluster 3 genes were enriched in IGF1R signaling cascade and insulin signaling pathways. The cluster analysis helped classify groups of functionally related proteins. Comprehending the biological processes of these functionally associated proteins will improve the understanding of the biological process of the PPI network as a whole [48,49,50].

Obesity and diabetes are major risk factors for each other as well as other diseases. The precise mechanisms linking the two complex polygenic diseases remain unclear. However, three hypotheses, namely “inflammation hypothesis”, “lipid overflow hypothesis”, and “adipokine hypothesis”, were proposed to elucidate such mechanisms [9]. In line with the “inflammation hypothesis”, we found that genes of clusters 1 and 3 are mainly involved with an insulin signaling pathway, IGF1R signaling cascade, and insulin receptor signaling cascade (Figure 5 and Figure 7). The progression of insulin resistance linked to obesity and T2D is attributed to inflammatory mechanisms in which the pro-inflammatory cytokines are produced by the abundance of adipose tissue. Such pro-inflammatory cytokines, tumor necrosis factor (TNF-α) and interleukin-6 (IL-6) are believed to link obesity to T2D through insulin resistance [51,52]. Overexpression of TNF-α in adipose tissue of obese individuals interferes with insulin resistance signaling via phosphorylating and converting IRS-1 to inhibit the proximal steps of insulin resistance signaling [53]. Islet amyloid polypeptide (IAPP) formed by islets of the pancreas can cause inflammasome NLR family pyrin domain containing 3 (NLRP3) in macrophages and dendritic cells to release IL-1β via its receptor on β-cells, which can signal cell death, impaired insulin secretory ability, and T2D. Obesity activates inflammasome NLRP3 and increases IL-1β through activation of caspase 1 and causes insulin resistance and reduction in fat oxidation. Saturated fatty acids and ceramide, associated with obesity and nutrient overload, can also trigger NLRP3 inflammasome to produce IL-1β that acts on the liver and impairs the activity of liver insulin, contributing to insulin resistance [54]. It has become clear that in the etiology of T2D, IL-1β is a crucial cytokine because both β-cell dysfunction and death have been implicated in this [55].

Accumulation of fat in tissues with inadequate storage capacity contributes to lipotoxicity in obese subjects, as stated by the “lipid overflow hypothesis”, eventually repressing insulin signaling transduction. Our data showed that genes of cluster 1 were enriched in FOXO-mediated transcription of oxidative stress and regulation of lipolysis in adipocytes. These genes were mainly involved in cholesterol metabolism, assembly–remodeling–clearance of plasma lipoprotein and chylomicron, metabolism of fat-soluble vitamins, LDL clearance, and HDL remodeling (Figure 5). The non-esterified fatty acids (NEFAs) synthesized in adipose tissues of obese individuals are believed to link insulin resistance with β-cell dysfunction [3]. Forkhead box protein O1 (*FOXO1*) transcription factor plays a crucial role in the protection of cells against oxidative stress. However, in tissues affected by diabetic complications, it promotes apoptosis and plays a destructive role [56]. In inflammatory signaling, *FOXO1* plays a collective role via NF-κB signaling. This cooperation couples pro-inflammatory cytokine production with insulin resistance and is thought to contribute to more significant inflammatory signaling in obesity and T2D.

*FOXO1* transcription factor also functions in the liver to incorporate hepatic insulin action to very low density lipoprotein (VLDL) production. In hepatic insulin resistance, the activity of *FOXO1* is augmented, leading to the overproduction of hepatic VLDL. Lipid metabolism abnormalities increase the risk of coronary artery disease in subjects with obesity and diabetes [57,58]. Disturbances in fat storage and mobilization are important factors that cause insulin resistance [59]. Storage and mobilization of fats involve chylomicron assembly, remodeling, and clearance involving *APOA1, APOA2, APOA5, APOB, APOE* genes, and the apolipoproteins assembly, remodeling, and clearance involving *APOA1, APOA2, APOA5, APOB, APOE, ALB,* and *PCSK9* genes. These apolipoproteins play a vital role in lipid homeostasis [60]. Therefore, excessive production of lipids, their storage, and mobilization to ectopic tissues contributes to obesity-associated insulin resistance and T2D. Healthy adipose tissue is characterized by the ability to expand passively to accommodate periods of excess nutrients. However, if the limit of adipose tissue expansion is reached, in adipose tissue, lipids could no longer be stored adequately and consequently “overflow” to other peripheral tissues, a phenomenon described by the “Lipid Overflow Hypothesis” [12,13]. Subcutaneous adipose tissue is considered the largest adipose tissue depot and the least biochemically harmful site for lipid conservation. Either by hypertrophic obesity (cell size increase) or hyperplastic obesity (new cell recruitment), this depot will extend. Unlike the hyperplastic response, which appears to protect against the dysfunction of subcutaneous adipose tissue, hypertrophic obesity is linked to an increased risk of T2D [61,62]. The storage of this ectopic fat in other non-subcutaneous adipose tissue is linked directly to the development of insulin resistance and T2D [63,64]. Therefore, accumulated fat in tissues, which is not ideal for lipid storage and, as a result, lipid compounds can pile up in those tissues that impede insulin signal transduction.

Our data showed that genes of clusters 1 and 2 are involved in the adipocytes regulatory function of lipolysis (Figure 5 and Figure 6), and genes of cluster 3 play a regulatory role in the insulin signaling pathway and lipolysis of adipocytes (Figure 7). In accordance with the “adipokine hypothesis”, in obese individuals, the excess of adipose tissue functions as an endocrine organ by releasing adipokines that result in insulin resistance and T2D development. The important adipokines include retinol-binding protein 4, adiponectin, vaspin (*SERPINA 12*), leptin, and the inflammatory chemokine (*CXCL 10*). Mutations in gene encoding leptin or the leptin receptor are reported to cause severe obesity, hyperphagia, and insulin resistance [65]. Our data showed that *ADRB1*, *ADRB2*, *ADRB3*, *CGA*, *GNAS*, *TSHR*, *INS*, *INSR*, and *IRS1* genes are involved in the diabesity PPI network (Figure 7). Polymorphisms in the three subtypes of β-adrenoceptor (*ADRB1*, *ADRB2,* and *ADRB3* genes) show a correlation with obesity and body weight-related disorders [66]. *ADRB1* also contributes to increased secretion of renin and ghrelin hormones, which are associated with T2D and insulin resistance [67]. Studies suggest that *p*.Arg389Gly polymorphism in the *ADRB1* may be a potential genetic biomarker to assess the risk of developing cardiovascular diseases [68]. Interestingly, many genes that are known to cause obesity are highly expressed in the central nervous system (CNS), which plays a crucial role in sensing and controlling the energy status of the body [69,70]. The imbalance of energy intake and energy expenditure in obesity is attributed to dysregulation of hypothalamic pathways [70]. One example is the melanocortin 4 receptor (MC4R) involved in the hypothalamic leptin–melanocortin signaling pathway that maintains energy homeostasis and is associated with food intake suppression. The standard form of monogenic obesity reported so far is the MC4R deficiency [71,72]. Our data showed that genes of cluster 1 are enriched in interleukin-10 signaling and interact with *CXCL8*, *CCL5*, *CCR2,* and *CCR5* (Figure 5). These genes are believed to contribute to the macrophage function in adipose tissue and insulin resistance in the “adipokine hypothesis”, which is the key trait of adipose cells (white) to act as an endocrine organ and to keep releasing a number of adipokines, which signal via paracrine and hormonal processes [73]. Inflammatory processes include most of these secreted molecules, including IL-1β, MCP-1, TNF-α, and IL-6, as described above. In obesity, the increasing adipose mass raises the circulating levels of these inflammatory markers and thus is believed to lead to insulin resistance and T2D progression. In vitro studies indicate that different inflammatory and oxidative stress factors suppress adiponectin expression [74]. In obese individuals, decreased levels of adiponectin and elevated levels of resistin are considered to signify the possibility of developing diabetes, even years before the advent of the condition [75].

Apart from our data that supports the three hypotheses of diabesity molecular mechanisms, our PPI network highlights the potential links to causal factors of metabolic complications in obesity. For instance, the *POMC* gene is involved in diabesity cluster 1 genes (Figure 4A). Studies of proopiomelanocortin (*POMC*) mutations showed an association between obesity in humans and a subsequent increase in the risk of obesity-related diseases, such as T1D and T2D [76,77]. The deficiency of glucose sensing by POMC neurons was observed in obese mice. This loss of glucose sensing by glucose-excited neurons was shown to involve a mitochondrial protein UCP2 (uncoupling protein 2) and is believed to have a role in T2D development [78]. Whereas the genes of cluster 1 include genes of the renin–angiotensin system (*RAS*) genes (Figure 4A and Figure 5). The *RAS* is involved in the regulation of fluid balance, blood pressure, and electrolyte [79]. *RAS* genes are widely expressed in adipose tissue and include angiotensinogen (*AGT*), angiotensin-converting enzyme (*ACE*), renin (*REN*), chymase (*CMA1*), type 1 angiotensin I receptor (*AGTR1*), and type 1 angiotensin II receptor (*AGTR2*) [80]. Adipose tissue RAS and systemic RAS are believed to be associated with obesity and insulin resistance, which might be a potential causal factor for metabolic complications in obesity [81,82].

Variations in any of these genes that are an integral part of the PPI network and pathways are known to be associated with diabesity (Figure 4A). The heritability of obesity can be explained not only by the variants of obesity and fat distribution but also by epigenetic marks. Extreme forms of obesity, such as Prader–Willi syndrome, are caused by imprinting failure. Environmental exposure affects the epigenetic profile during critical growth periods and has been persuasively linked to obesity susceptibility [83]. Only obese individuals susceptible to insulin secretion deficiencies would develop diabetes because insulin resistance alone cannot trigger diabetes. This predisposition is resolved by genetic and environmental factors [84]. Therefore, obesity may incite diabetes as an epigenetic phenomenon in genetically predisposed individuals [85]. Current efforts to combat obesity through exercise, diet, and surgery are largely ineffective in providing long-term, sustainable solutions. The inability to understand the pathophysiology of obesity and T2D makes the development of therapeutics and preventive strategies challenging. Identification of genetic biomarkers involved in disease predisposition may help explain the pathogenesis of the disease and provide opportunities for personalized medicine [86].

## 5. Conclusions

Diabesity is considered as a global health concern that necessitates the understanding of its molecular pathology. Excessive secretion of adipokines in obesity plays a critical role in T1D, T2D, and MODY. In addition, diabetes results in organ dysfunction, including endocrine, respiratory, and liver, and immune dysfunction is exacerbated by the production of inflammatory adipokines. Our study specifically focused on unraveling the molecular etiology of obesity-associated diabetes genes by demonstrating the interconnected pathways of diabesity-related signaling cascades. Established signaling cascades from our study provide an interplay between the genetic links of diabesity, which resulted in patients with long-term diabetic complications. We found the central signaling cascades, such as adipocytes regulatory function of lipolysis, FOXO-mediated transcription of oxidative stress, regulation of lipolysis in adipocytes, insulin receptor signaling pathway, and IGF1R signaling cascade, are involved in the pathogenesis of diabesity. The findings of this study will help identify potential novel genetic biomarkers for clinical molecular diagnosis of the disease. Moreover, this study serves as a potential platform for the discovery of future therapeutic drug targets.

## Figures and Tables

**Table 1:** Network statistics of string interactions from the Cytoscape plugin (NetworkAnalyzer) for the identified obesity genes associated with type-1 diabetes (T1D), type-2 diabetes (T2D), and maturity-onset diabetes of the young (MODY).

S.no	Simple Parameters	Comprehended Values
1.	Nodes number	514
2.	Characteristic path length	3.171
3.	Network heterogeneity	1.068
4.	Clustering coefficient	0.484
5.	Average number of neighbors	16.054
6.	Network centralization	0.196
7.	Network density	0.031
8.	Network diameter	9

**Table 2:** The most densely interconnected regions of the protein-protein interactions (PPI) network are categorized by MCODE from our identified associated genes between Obesity, T1D, T2D, and MODY.

Cluster	Score (Density * # Nodes)	Nodes	Edges	Nodes IDs
1	33.61	83	1378	*C3, TNC, APOA2, MTNR1A, MTNR1B, CXCL5, SERPINA1, AHSG, ALB, GAST, BMP4, SPP1, SST, APOA5, PYY, F5, FGF23, IGFBP3, PPY, CCK, TRH, CCKAR, GNRH1, GCG, GRP, OPRM1, HCRT, GPR39, GHSR, IGFBP7, DRD4, POMC, CX3CR1, ADRA2A, APOA1, TAC1, NTS, GAL, ADRA2B, TIMP1, HTR1A, CCR2, APOB, FGA, BDKRB2, GNAI1, KNG1, PNPLA2, APOE, APLNR, APLN, IGFBP1, CASR, AGTR1, CXCL8, EDN1, MLN, EDNRA, PROC, OXT, PCSK9, **AGT**, CNR1, DRD2, PIK3R1, PIK3CA, F2, CXCR4, SERPINC1, IGFBP5, CCL5, APP, HTR2A, CCR5, CSF1, CNR2, NPY, GCGR, CP, TF, IL6, CST3, ADORA1*
2	16.788	34	277	*GIP, PROS1, **FGB**, TGFB1, CLU, ADRB3, IGF1, MC4R, VWF, A2M, CALCA, PTH, IAPP, HGF, CGA, RAMP1, ADRB1, DRD1, F13A1, SERPINF2, SPARC, TSHR, GNAS, GLP1R, THBS1, GPBAR1, ADRB2, SERPINE1, ADM, CRH, MC3R, GHRH, IGF2, VEGFA*
3	6.783	24	78	*SH3GL2, INPPL1, TIMP2, CTSS, IL5, LTF, SHC1, PIK3C2A, IRS1, TFRC, MMP8, PTPN1, HP, B2M, **LDLR**, PRKCZ, MMP9, INS, WNT5A, FTH1, INSR, IGF1R, IGF2R, RPS27A*

* Network computation based on node score cutoff and K-core; Cluster seeds are in bold.

## Supplementary Materials

The following are available online at https://www.mdpi.com/2073-4425/11/11/1256/s1, Table S1: The most significant obesity genes that are common between the different types of diabetes; T1D, T2D, and MODY, Table S2: Involved GO terms from ClueGO enrichment analysis for cluster 1 genes., Table S3: Involved GO terms from ClueGO enrichment analysis for cluster 2 genes., Table S4: Involved GO terms from ClueGO enrichment analysis for cluster 3 genes.
