# Supplementary material for: Involvement of Essential Signaling Cascades and Analysis of Gene Networks in Diabesity
Source: Genes (Basel). 2020 Oct 25;11(11):1256. doi: 10.3390/genes11111256 (PMC7693799; doi:10.3390/genes11111256)
Supplement: Supplementary file 1 [file genes-11-01256-s001.pdf]

## Supplementary materials

**Supplementary Table 1.** The most significant obesity genes that are common between the different types of diabetes; T1D, T2D, and MODY.

| Genes | Genes | Genes | Genes |
|---|---|---|---|
| *VDR* | *BDNF* | *PPARGC1A* | *SERPINC1* |
| *ACE* | *IFNG* | *SERPINE1* | *APOA1* |
| *HMGCR* | *ABCA1* | *CYP19A1* | *CCK* |
| *MMP2* | *RETN* | *VCAM1* | *ADRB2* |
| *EDN1* | *IL4* | *TP53* | *GAPDH* |
| *ABCB7* | *BCHE* | *TNF* | *APC* |
| *GSR* | *BCL2* | *INSR* | *TLR2* |
| *FASLG* | *TNFRSF11B* | *IFNA1* | *VEGFA* |
| *GGT1* | *CD36* | *HNF1B* | *CDKN2A* |
| *CYBA* | *FGF2* | *GNRH1* | *TGFB1* |
| *IGF1* | *CLU* | *HLA-DRB1* | *KCNJ11* |
| *CCL11* | *PCK2* | *IL1A* | *TFRC* |
| *PTEN* | *HK2* | *LDLR* | *ABCC8* |
| *HMOX1* | *G6PD* | *LPL* | *GAST* |
| *HNF1A* | *TF* | *CAT* | *TSHR* |
| *NOS2* | *GCK* | *MPO* | *JUN* |
| *INS* | *IGF1R* | *TCF7L2* | *HCRT* |
| *FLT1* | *IRS1* | *LIPC* | *SERPINF2* |
| *SLC6A4* | *GHRL* | *SELP* | *TIMP1* |
| *SOD2* | *ICAM1* | *GCG* | *CSF2* |
| *BGLAP* | *RECK* | *F5* | *GHRH* |
| *MMP3* | *RPS27A* | *IL8* | *MMP9* |
| *PPARA* | *TNFRSF1B* | *HLA-A* | *CD14* |
| *NGF* | *APOB* | *SLC2A2* | *ADIPOQ* |
| *GATA3* | *CS* | *AKT1* | |
| *IL2RA* | *LTF* | *PIK3R1* | |
| *PAX4* | *IL5* | *GRP* | |
| *TTR* | *AGT* | *NR1H2* | |
| *LEP* | *CGA* | *TNFRSF1A* | |
| *PTPRN2* | *PPARG* | *STAT3* | |
| *OXT* | *HNF4A* | *SPP1* | |
| *CYP1A2* | *CRP* | *F2* | |
| *TG* | *HIF1A* | *PTGS2* | |
| *CYP2C19* | *CALCA* | *AHSG* | |
| *NOS1* | *SERPINA1* | *B2M* | |
| *CRH* | *NOS3* | *LRP5* | |
| *IL10* | *MAPT* | *OLR1* | |
| *PRL* | *GLP1R* | *GH1* | |
| *IGF2* | *CASR* | *ITGB3* | |
| *IAPP* | *CNR1* | *CYP2C9* | |

| | | | |
|---|---|---|---|
| *MTHFR* | *DKK1* | *IL1B* | |
| *ISL1* | *NFE2L2* | *REN* | |
| *PAX6* | *NR3C1* | *VWF* | |
| *TNFSF11* | *HP* | *NPY* | |
| *GHR* | *OPRM1* | *IL18* | |
| *DPP4* | *CYP17A1* | *GAD2* | |
| *NPPB* | *TLR4* | *IGFBP2* | |
| *APOC3* | *LEPR* | *AR* | |
| *SOD1* | *BMP6* | *GAD1* | |
| *IFNA2* | *NPPA* | *PCSK9* | |
| *CD79A* | *ESR1* | *EPO* | |
| *FOXM1* | *SST* | *ACHE* | |
| *PIK3CA* | *PRF1* | *CP* | |
| *APOE* | *BMP4* | *KLK3* | |
| *IL2* | *NEUROD1* | *IL1R1* | |
| *FAS* | *SPARC* | *CCL2* | |
| *C3* | *AGTR1* | *HGF* | |
| *PDX1* | *IGFBP3* | *IL6* | |
| *PON1* | *ALB* | *APP* | |
| *NEUROG3* | *PTGS1* | *CPT2* | |
| *FOXA2* | *LCAT* | *COMT* | |
| *IGFBP1* | *APOA4* | *UCP2* | |
| *HSPD1* | *POMC* | *PROC* | |

**Supplementary Table 2.** Involved GO terms from ClueGo enrichment analysis for cluster 1 genes.

| GO ID | GO Term | Group p-Value Corrected with Bonferroni step down | % Associated Genes | Nr. Genes | Associated Genes Found |
|---|---|---|---|---|---|
| **R-HSA:375276** | Peptide ligand-binding receptors | 7.15293E-47 | 18.42 | 35.00 | *[AGT, AGTR1, APLN, APLNR, APP, BDKRB2, C3, CCK, CCKAR, CCL5, CCR2, CCR5, CX3CR1, CXCL5, CXCL8, CXCR4, EDN1, EDNRA, F2, GAL, GHSR, GRP, HCRT, KNG1, MLN, NPY, NTS, OPRM1, OXT, POMC, PPY, PYY, SST, TAC1, TRH]* |
| **R-HSA:373076** | Class A/1 (Rhodopsin-like receptors) | 7.15293E-47 | 14.81 | 48.00 | *[ADORA1, ADRA2A, ADRA2B, AGT, AGTR1, APLN, APLNR, APP, BDKRB2, C3, CCK, CCKAR, CCL5, CCR2, CCR5, CNR1, CNR2, CX3CR1, CXCL5, CXCL8, CXCR4, DRD2, DRD4, EDN1, EDNRA, F2, GAL, GHSR, GNRH1, GPR39, GRP, HCRT, HTR1A, HTR2A, KNG1, MLN, MTNR1A, MTNR1B, NPY, NTS, OPRM1, OXT, POMC, PPY, PYY, SST, TAC1, TRH]* |
| **KEGG:04080** | Neuroactive ligand-receptor interaction | 7.15293E-47 | 12.13 | 41.00 | *[ADORA1, ADRA2A, ADRA2B, AGT, AGTR1, APLN, APLNR, BDKRB2, C3, CCK, CCKAR, CNR1, CNR2, DRD2, DRD4, EDN1, EDNRA, F2, GAL, GCG, GCGR, GHSR, GNRH1, GRP, HCRT, HTR1A, HTR2A, KNG1, MLN, MTNR1A, MTNR1B, NPY, NTS, OPRM1, OXT, POMC, PPY, PYY, SST, TAC1, TRH]* |
| **R-HSA:500792** | GPCR ligand binding | 7.15293E-47 | 11.16 | 51.00 | *[ADORA1, ADRA2A, ADRA2B, AGT, AGTR1, APLN, APLNR, APP, BDKRB2, C3, CASR, CCK, CCKAR, CCL5, CCR2, CCR5, CNR1, CNR2, CX3CR1, CXCL5, CXCL8, CXCR4, DRD2, DRD4, EDN1, EDNRA, F2, GAL, GCG, GCGR, GHSR, GNRH1, GPR39, GRP, HCRT, HTR1A, HTR2A, KNG1, MLN, MTNR1A, MTNR1B, NPY, NTS, OPRM1, OXT, POMC, PPY, PYY, SST, TAC1, TRH]* |
| **R-HSA:418594** | G alpha (i) signalling events | 7.15293E-47 | 9.09 | 37.00 | *[ADORA1, ADRA2A, ADRA2B, AGT, APLN, APLNR, APOA1, APOA2, APOB, APOE, APP, BDKRB2, C3, CASR, CCL5, CCR2, CCR5, CNR1, CNR2, CX3CR1, CXCL5, CXCL8, CXCR4, DRD2, DRD4, GAL, GNAI1, HTR1A, KNG1, MTNR1A, MTNR1B, NPY, OPRM1, POMC, PPY, PYY, SST]* |
| **R-HSA:8957275** | Post-translational protein phosphorylation | 7.24486E-42 | 28.70 | 31.00 | *[AHSG, ALB, APOA1, APOA2, APOA5, APOB, APOE, APP, BMP4, C3, CP, CSF1, CST3, F5, FGA, FGF23, IGFBP1, IGFBP3, IGFBP5, IGFBP7, IL6, KNG1, PCSK9, PNPLA2, PROC, SERPINA1, SERPINC1, SPP1, TF, TIMP1, TNC]* |
| **R-HSA:381426** | Regulation of Insulin-like Growth Factor (IGF) transport and uptake by | 7.24486E-42 | 25.60 | 32.00 | *[AHSG, ALB, APOA1, APOA2, APOA5, APOB, APOE, APP, BMP4, C3, CP, CSF1, CST3, F2, F5, FGA, FGF23, IGFBP1, IGFBP3,* |

| | | | | | |
|---|---|---|---|---|---|
| | Insulin-like Growth Factor Binding Proteins (IGFBPs) | | | | *IGFBP5, IGFBP7, IL6, KNG1, PCSK9, PNPLA2, PROC, SERPINA1, SERPINC1, SPP1, TF, TIMP1, TNC]* |
| **R-HSA:416476** | G alpha (q) signalling events | 6.88134E-26 | 12.44 | 27.00 | *[AGT, AGTR1, APP, BDKRB2, CASR, CCK, CCKAR, EDN1, EDNRA, F2, GAST, GCG, GCGR, GHSR, GNRH1, GPR39, GRP, HCRT, HTR2A, KNG1, MLN, NTS, OXT, PIK3CA, PIK3R1, TAC1, TRH]* |
| **R-HSA:392023** | Adrenaline signalling through Alpha-2 adrenergic receptor | 6.72026E-10 | 66.67 | 2.00 | *[ADRA2A, ADRA2B]* |
| **R-HSA:390651** | Dopamine receptors | 6.72026E-10 | 40.00 | 2.00 | *[DRD2, DRD4]* |
| **R-HSA:140875** | Common Pathway of Fibrin Clot Formation | 6.72026E-10 | 22.73 | 5.00 | *[F2, F5, FGA, PROC, SERPINC1]* |
| **R-HSA:390696** | Adrenoceptors | 6.72026E-10 | 22.22 | 2.00 | *[ADRA2A, ADRA2B]* |
| **R-HSA:390666** | Serotonin receptors | 6.72026E-10 | 16.67 | 2.00 | *[HTR1A, HTR2A]* |
| **R-HSA:375280** | Amine ligand-binding receptors | 6.72026E-10 | 14.29 | 6.00 | *[ADRA2A, ADRA2B, DRD2, DRD4, HTR1A, HTR2A]* |
| **R-HSA:76009** | Platelet Aggregation (Plug Formation) | 6.72026E-10 | 10.26 | 4.00 | *[ADRA2A, ADRA2B, F2, FGA]* |
| **R-HSA:140875** | Common Pathway of Fibrin Clot Formation | 1.1004E-08 | 22.73 | 5.00 | *[F2, F5, FGA, PROC, SERPINC1]* |
| **R-HSA:159763** | Transport of gamma-carboxylated protein precursors from the endoplasmic reticulum to the Golgi apparatus | 1.1004E-08 | 22.22 | 2.00 | *[F2, PROC]* |
| **R-HSA:159740** | Gamma-carboxylation of protein precursors | 1.1004E-08 | 20.00 | 2.00 | *[F2, PROC]* |
| **R-HSA:159782** | Removal of aminoterminal propeptides from gamma-carboxylated proteins | 1.1004E-08 | 20.00 | 2.00 | *[F2, PROC]* |
| **R-HSA:140837** | Intrinsic Pathway of Fibrin Clot Formation | 1.1004E-08 | 18.18 | 4.00 | *[F2, KNG1, PROC, SERPINC1]* |
| **R-HSA:159854** | Gamma-carboxylation, transport, and amino-terminal cleavage of proteins | 1.1004E-08 | 18.18 | 2.00 | *[F2, PROC]* |

| | | | | | |
|---|---|---|---|---|---|
| **R-HSA:140877** | Formation of Fibrin Clot (Clotting Cascade) | 1.1004E-08 | 15.38 | 6.00 | *[F2, F5, FGA, KNG1, PROC, SERPINC1]* |
| **KEGG:04610** | Complement and coagulation cascades | 1.1004E-08 | 11.39 | 9.00 | *[BDKRB2, C3, F2, F5, FGA, KNG1, PROC, SERPINA1, SERPINC1]* |
| **R-HSA:76009** | Platelet Aggregation (Plug Formation) | 1.1004E-08 | 10.26 | 4.00 | *[ADRA2A, ADRA2B, F2, FGA]* |
| **R-HSA:173107** | Binding and entry of HIV virion | 2.31219E-08 | 20.00 | 2.00 | *[CCR5, CXCR4]* |
| **R-HSA:6783783** | Interleukin-10 signaling | 2.31219E-08 | 14.89 | 7.00 | *[CCL5, CCR2, CCR5, CSF1, CXCL8, IL6, TIMP1]* |
| **R-HSA:380108** | Chemokine receptors bind chemokines | 2.31219E-08 | 14.58 | 7.00 | *[CCL5, CCR2, CCR5, CX3CR1, CXCL5, CXCL8, CXCR4]* |
| **R-HSA:162594** | Early Phase of HIV Life Cycle | 2.31219E-08 | 10.00 | 2.00 | *[CCR5, CXCR4]* |
| **KEGG:05142** | Chagas disease (American trypanosomiasis) | 3.50978E-07 | 8.74 | 9.00 | *[BDKRB2, C3, CCL5, CXCL8, GNAI1, IL6, KNG1, PIK3CA, PIK3R1]* |
| **KEGG:04614** | Renin-angiotensin system | 0.000272724 | 8.70 | 2.00 | *[AGT, AGTR1]* |
| **KEGG:04924** | Renin secretion | 0.000272724 | 8.70 | 6.00 | *[ADORA1, AGT, AGTR1, EDN1, EDNRA, GNAI1]* |
| **R-HSA:8963901** | Chylomicron remodeling | 0.000437865 | 55.56 | 5.00 | *[APOA1, APOA2, APOA5, APOB, APOE]* |
| **R-HSA:8963888** | Chylomicron assembly | 0.000437865 | 44.44 | 4.00 | *[APOA1, APOA2, APOB, APOE]* |
| **R-HSA:8964026** | Chylomicron clearance | 0.000437865 | 40.00 | 2.00 | *[APOB, APOE]* |
| **R-HSA:3000471** | Scavenging by Class B Receptors | 0.000437865 | 33.33 | 2.00 | *[APOA1, APOB]* |
| **R-HSA:8964058** | HDL remodeling | 0.000437865 | 30.00 | 3.00 | *[ALB, APOA1, APOE]* |
| **R-HSA:8963898** | Plasma lipoprotein assembly | 0.000437865 | 22.22 | 4.00 | *[APOA1, APOA2, APOB, APOE]* |
| **R-HSA:8963899** | Plasma lipoprotein remodeling | 0.000437865 | 20.00 | 6.00 | *[ALB, APOA1, APOA2, APOA5, APOB, APOE]* |
| **R-HSA:3000480** | Scavenging by Class A Receptors | 0.000437865 | 15.79 | 3.00 | *[APOA1, APOB, APOE]* |
| **R-HSA:2168880** | Scavenging of heme from plasma | 0.000437865 | 15.38 | 2.00 | *[ALB, APOA1]* |
| **R-HSA:8964043** | Plasma lipoprotein clearance | 0.000437865 | 12.12 | 4.00 | *[APOA1, APOB, APOE, PCSK9]* |
| **R-HSA:5686938** | Regulation of TLR by endogenous ligand | 0.000437865 | 10.53 | 2.00 | *[APOB, FGA]* |

| | | | | | |
|---|---|---|---|---|---|
| **R-HSA:8964038** | LDL clearance | 0.000437865 | 10.53 | 2.00 | *[APOB, PCSK9]* |
| **R-HSA:174824** | Plasma lipoprotein assembly, remodeling, and clearance | 0.000437865 | 10.14 | 7.00 | *[ALB, APOA1, APOA2, APOA5, APOB, APOE, PCSK9]* |
| **KEGG:04979** | Cholesterol metabolism | 0.000437865 | 10.00 | 5.00 | *[APOA1, APOA2, APOB, APOE, PCSK9]* |
| **R-HSA:2173782** | Binding and Uptake of Ligands by Scavenger Receptors | 0.000437865 | 9.52 | 4.00 | *[ALB, APOA1, APOB, APOE]* |
| **R-HSA:975634** | Retinoid metabolism and transport | 0.000437865 | 9.30 | 4.00 | *[APOA1, APOA2, APOB, APOE]* |
| **R-HSA:6806667** | Metabolism of fat-soluble vitamins | 0.000437865 | 8.51 | 4.00 | *[APOA1, APOA2, APOB, APOE]* |
| **KEGG:04977** | Vitamin digestion and absorption | 0.000437865 | 8.33 | 2.00 | *[APOA1, APOB]* |
| **R-HSA:8851907** | MET activates PI3K/AKT signaling | 0.004786962 | 33.33 | 2.00 | *[PIK3CA, PIK3R1]* |
| **R-HSA:9603381** | Activated NTRK3 signals through PI3K | 0.004786962 | 33.33 | 2.00 | *[PIK3CA, PIK3R1]* |
| **R-HSA:9028335** | Activated NTRK2 signals through PI3K | 0.004786962 | 28.57 | 2.00 | *[PIK3CA, PIK3R1]* |
| **R-HSA:198203** | PI3K/AKT activation | 0.004786962 | 22.22 | 2.00 | *[PIK3CA, PIK3R1]* |
| **R-HSA:1250342** | PI3K events in ERBB4 signaling | 0.004786962 | 20.00 | 2.00 | *[PIK3CA, PIK3R1]* |
| **R-HSA:8853334** | Signaling by FGFR3 fusions in cancer | 0.004786962 | 20.00 | 2.00 | *[PIK3CA, PIK3R1]* |
| **R-HSA:5655291** | Signaling by FGFR4 in disease | 0.004786962 | 18.18 | 2.00 | *[PIK3CA, PIK3R1]* |
| **R-HSA:5654710** | PI-3K cascade:FGFR3 | 0.004786962 | 16.67 | 3.00 | *[FGF23, PIK3CA, PIK3R1]* |
| **R-HSA:9027276** | Erythropoietin activates Phosphoinositide-3-kinase (PI3K) | 0.004786962 | 16.67 | 2.00 | *[PIK3CA, PIK3R1]* |
| **R-HSA:5654720** | PI-3K cascade:FGFR4 | 0.004786962 | 15.00 | 3.00 | *[FGF23, PIK3CA, PIK3R1]* |
| **R-HSA:5654689** | PI-3K cascade:FGFR1 | 0.004786962 | 14.29 | 3.00 | *[FGF23, PIK3CA, PIK3R1]* |
| **R-HSA:5655332** | Signaling by FGFR3 in disease | 0.004786962 | 13.64 | 3.00 | *[FGF23, PIK3CA, PIK3R1]* |

| | | | | | |
|---|---|---|---|---|---|
| **R-HSA:8853338** | Signaling by FGFR3 point mutants in cancer | 0.004786962 | 13.64 | 3.00 | *[FGF23, PIK3CA, PIK3R1]* |
| **R-HSA:5637810** | Constitutive Signaling by EGFRvIII | 0.004786962 | 13.33 | 2.00 | *[PIK3CA, PIK3R1]* |
| **R-HSA:5637812** | Signaling by EGFRvIII in Cancer | 0.004786962 | 13.33 | 2.00 | *[PIK3CA, PIK3R1]* |
| **R-HSA:5654695** | PI-3K cascade:FGFR2 | 0.004786962 | 13.04 | 3.00 | *[FGF23, PIK3CA, PIK3R1]* |
| **R-HSA:1963642** | PI3K events in ERBB2 signaling | 0.004786962 | 12.50 | 2.00 | *[PIK3CA, PIK3R1]* |
| **R-HSA:2730905** | Role of LAT2/NTAL/LAB on calcium mobilization | 0.004786962 | 12.50 | 2.00 | *[PIK3CA, PIK3R1]* |
| **R-HSA:5654708** | Downstream signaling of activated FGFR3 | 0.004786962 | 12.00 | 3.00 | *[FGF23, PIK3CA, PIK3R1]* |
| **R-HSA:180292** | GAB1 signalosome | 0.004786962 | 11.76 | 2.00 | *[PIK3CA, PIK3R1]* |
| **R-HSA:9034015** | Signaling by NTRK3 (TRKC) | 0.004786962 | 11.76 | 2.00 | *[PIK3CA, PIK3R1]* |
| **R-HSA:1839117** | Signaling by cytosolic FGFR1 fusion mutants | 0.004786962 | 11.11 | 2.00 | *[PIK3CA, PIK3R1]* |
| **R-HSA:210993** | Tie2 Signaling | 0.004786962 | 11.11 | 2.00 | *[PIK3CA, PIK3R1]* |
| **R-HSA:5654716** | Downstream signaling of activated FGFR4 | 0.004786962 | 11.11 | 3.00 | *[FGF23, PIK3CA, PIK3R1]* |
| **KEGG:04923** | Regulation of lipolysis in adipocytes | 0.004786962 | 10.91 | 6.00 | *[ADORA1, GNAI1, NPY, PIK3CA, PIK3R1, PNPLA2]* |
| **R-HSA:1236382** | Constitutive Signaling by Ligand-Responsive EGFR Cancer Variants | 0.004786962 | 10.53 | 2.00 | *[PIK3CA, PIK3R1]* |
| **R-HSA:5637815** | Signaling by Ligand-Responsive EGFR Variants in Cancer | 0.004786962 | 10.53 | 2.00 | *[PIK3CA, PIK3R1]* |
| **R-HSA:5654696** | Downstream signaling of activated FGFR2 | 0.004786962 | 10.00 | 3.00 | *[FGF23, PIK3CA, PIK3R1]* |
| **R-HSA:1839124** | FGFR1 mutant receptor activation | 0.004786962 | 9.68 | 3.00 | *[FGF23, PIK3CA, PIK3R1]* |
| **R-HSA:5654687** | Downstream signaling of activated FGFR1 | 0.004786962 | 9.68 | 3.00 | *[FGF23, PIK3CA, PIK3R1]* |
| **R-HSA:389357** | CD28 dependent PI3K/Akt signaling | 0.004786962 | 9.09 | 2.00 | *[PIK3CA, PIK3R1]* |

| | | | | | |
|---|---|---|---|---|---|
| **R-HSA:912631** | Regulation of signaling by CBL | 0.004786962 | 9.09 | 2.00 | *[PIK3CA, PIK3R1]* |
| **R-HSA:373753** | Nephrin family interactions | 0.004786962 | 8.70 | 2.00 | *[PIK3CA, PIK3R1]* |
| **R-HSA:1643713** | Signaling by EGFR in Cancer | 0.004786962 | 8.00 | 2.00 | *[PIK3CA, PIK3R1]* |
| **R-HSA:2029485** | Role of phospholipids in phagocytosis | 0.004786962 | 8.00 | 2.00 | *[PIK3CA, PIK3R1]* |
| **R-HSA:9006115** | Signaling by NTRK2 (TRKB) | 0.004786962 | 8.00 | 2.00 | *[PIK3CA, PIK3R1]* |
| **R-HSA:9006335** | Signaling by Erythropoietin | 0.004786962 | 8.00 | 2.00 | *[PIK3CA, PIK3R1]* |
| **R-HSA:9615017** | FOXO-mediated transcription of oxidative stress, metabolic and neuronal genes | 0.005339022 | 10.34 | 3.00 | *[IGFBP1, NPY, POMC]* |
| **R-HSA:202040** | G-protein activation | 0.005773145 | 10.71 | 3.00 | *[GNAI1, OPRM1, POMC]* |
| **KEGG:05143** | African trypanosomiasis | 0.006542366 | 8.11 | 3.00 | *[APOA1, IL6, KNG1]* |
| **R-HSA:380994** | ATF4 activates genes | 0.013149546 | 8.00 | 2.00 | *[CXCL8, IGFBP1]* |
| **R-HSA:381771** | Synthesis, secretion, and inactivation of Glucagon-like Peptide-1 (GLP-1) | 0.022380594 | 10.00 | 2.00 | *[GCG, GRP]* |
| **R-HSA:400508** | Incretin synthesis, secretion, and inactivation | 0.022380594 | 8.70 | 2.00 | *[GCG, GRP]* |

**Supplementary Table 3.** Involved GO terms from ClueGo enrichment analysis for cluster 2 genes.

| GO ID | GO Term | Term p-Value Corrected with Bonferroni step down | % Associated Genes | Nr. Genes | Associated Genes Found |
|---|---|---|---|---|---|
| **R-HSA:114608** | Platelet degranulation | 3.48546E-20 | 11.63 | 15.00 | *[A2M, CLU, F13A1, FGB, HGF, IGF1, IGF2, PROS1, SERPINE1, SERPINF2, SPARC, TGFB1, THBS1, VEGFA, VWF]* |

| | | | | | |
|---|---|---|---|---|---|
| **R-HSA:76005** | Response to elevated platelet cytosolic Ca2+ | 5.87143E-20 | 11.19 | 15.00 | *[A2M, CLU, F13A1, FGB, HGF, IGF1, IGF2, PROS1, SERPINE1, SERPINF2, SPARC, TGFB1, THBS1, VEGFA, VWF]* |
| **R-HSA:373080** | Class B/2 (Secretin family receptors) | 9.2479E-13 | 10.53 | 10.00 | *[ADM, CALCA, CRH, GHRH, GIP, GLP1R, GNAS, IAPP, PTH, RAMP1]* |
| **KEGG:04610** | Complement and coagulation cascades | 4.78905E-10 | 10.13 | 8.00 | *[A2M, CLU, F13A1, FGB, PROS1, SERPINE1, SERPINF2, VWF]* |
| **KEGG:04923** | Regulation of lipolysis in adipocytes | 9.30048E-08 | 10.91 | 6.00 | *[ADRB1, ADRB2, ADRB3, CGA, GNAS, TSHR]* |
| **R-HSA:419812** | Calcitonin-like ligand receptors | 1.06302E-07 | 40.00 | 4.00 | *[ADM, CALCA, IAPP, RAMP1]* |
| **R-HSA:140877** | Formation of Fibrin Clot (Clotting Cascade) | 6.20737E-07 | 12.82 | 5.00 | *[A2M, F13A1, FGB, PROS1, VWF]* |
| **R-HSA:390696** | Adrenoceptors | 1.26179E-05 | 33.33 | 3.00 | *[ADRB1, ADRB2, ADRB3]* |
| **R-HSA:420092** | Glucagon-type ligand receptors | 1.31787E-05 | 12.12 | 4.00 | *[GHRH, GIP, GLP1R, GNAS]* |
| **R-HSA:140875** | Common Pathway of Fibrin Clot Formation | 0.000161063 | 13.64 | 3.00 | *[F13A1, FGB, PROS1]* |
| **R-HSA:8866910** | TFAP2 (AP-2) family regulates transcription of growth factors and their receptors | 0.00082292 | 13.33 | 2.00 | *[CGA, VEGFA]* |
| **R-HSA:354194** | GRB2:SOS provides linkage to MAPK signaling for Integrins | 0.00082292 | 13.33 | 2.00 | *[FGB, VWF]* |
| **R-HSA:372708** | p130Cas linkage to MAPK signaling for integrins | 0.00082292 | 13.33 | 2.00 | *[FGB, VWF]* |
| **R-HSA:2428933** | SHC-related events triggered by IGF1R | 0.001140851 | 22.22 | 2.00 | *[IGF1, IGF2]* |
| **R-HSA:75205** | Dissolution of Fibrin Clot | 0.001227039 | 15.38 | 2.00 | *[SERPINE1, SERPINF2]* |
| **R-HSA:375281** | Hormone ligand-binding receptors | 0.001560206 | 16.67 | 2.00 | *[CGA, TSHR]* |

**Supplementary Table 4.** Involved GO terms from ClueGo enrichment analysis for cluster 3 genes.

| GO ID | GO Term | Term p-Value Corrected with Bonferroni step down | % Associated Genes | Nr. Genes | Associated Genes Found |
|---|---|---|---|---|---|
| **R-HSA:74749** | Signal attenuation | 1.00541E-07 | 40.00 | 4.00 | *[INS, INSR, IRS1, SHC1]* |
| **R-HSA:74749** | Signal attenuation | 1.00541E-07 | 40.00 | 4.00 | *[INS, INSR, IRS1, SHC1]* |

| | | | | | |
|---|---|---|---|---|---|
| **KEGG:04910** | Insulin signaling pathway | 2.53779E-07 | 5.11 | 7.00 | *[INPPL1, INS, INSR, IRS1, PRKCZ, PTPN1, SHC1]* |
| **R-HSA:8856825** | Cargo recognition for clathrin-mediated endocytosis | 1.85804E-06 | 5.66 | 6.00 | *[IGF2R, LDLR, RPS27A, SH3GL2, TFRC, WNT5A]* |
| **R-HSA:74713** | IRS activation | 2.49046E-06 | 60.00 | 3.00 | *[INS, INSR, IRS1]* |
| **R-HSA:432722** | Golgi Associated Vesicle Biogenesis | 2.59797E-06 | 8.93 | 5.00 | *[FTH1, IGF2R, PIK3C2A, SH3GL2, TFRC]* |
| **R-HSA:199992** | trans-Golgi Network Vesicle Budding | 9.03152E-06 | 6.94 | 5.00 | *[FTH1, IGF2R, PIK3C2A, SH3GL2, TFRC]* |
| **R-HSA:421837** | Clathrin derived vesicle budding | 9.03152E-06 | 6.94 | 5.00 | *[FTH1, IGF2R, PIK3C2A, SH3GL2, TFRC]* |
| **KEGG:04930** | Type II diabetes mellitus | 6.23421E-05 | 8.70 | 4.00 | *[INS, INSR, IRS1, PRKCZ]* |
| **R-HSA:512988** | Interleukin-3, Interleukin-5 and GM-CSF signaling | 7.17434E-05 | 8.33 | 4.00 | *[IL5, INPPL1, RPS27A, SHC1]* |
| **KEGG:04913** | Ovarian steroidogenesis | 7.54143E-05 | 8.16 | 4.00 | *[IGF1R, INS, INSR, LDLR]* |
| **R-HSA:74751** | Insulin receptor signalling cascade | 0.00010795 | 7.41 | 4.00 | *[INS, INSR, IRS1, SHC1]* |
| **R-HSA:74751** | Insulin receptor signalling cascade | 0.00010795 | 7.41 | 4.00 | *[INS, INSR, IRS1, SHC1]* |
| **KEGG:04213** | Longevity regulating pathway | 0.000181635 | 6.45 | 4.00 | *[IGF1R, INS, INSR, IRS1]* |
| **R-HSA:6807004** | Negative regulation of MET activity | 0.00024795 | 14.29 | 3.00 | *[PTPN1, RPS27A, SH3GL2]* |
| **R-HSA:74752** | Signaling by Insulin receptor | 0.000420816 | 5.13 | 4.00 | *[INS, INSR, IRS1, SHC1]* |
| **R-HSA:74752** | Signaling by Insulin receptor | 0.000420816 | 5.13 | 4.00 | *[INS, INSR, IRS1, SHC1]* |
| **R-HSA:6806834** | Signaling by MET | 0.000424953 | 5.06 | 4.00 | *[PTPN1, RPS27A, SH3GL2, SHC1]* |
| **R-HSA:912526** | Interleukin receptor SHC signaling | 0.000478543 | 11.11 | 3.00 | *[IL5, INPPL1, SHC1]* |
| **R-HSA:1592389** | Activation of Matrix Metalloproteinases | 0.000847019 | 9.09 | 3.00 | *[MMP8, MMP9, TIMP2]* |
| **KEGG:04960** | Aldosterone-regulated sodium reabsorption | 0.001145307 | 8.11 | 3.00 | *[INS, INSR, IRS1]* |
| **R-HSA:451927** | Interleukin-2 family signaling | 0.001841966 | 6.82 | 3.00 | *[IL5, INPPL1, SHC1]* |
| **R-HSA:177929** | Signaling by EGFR | 0.002418122 | 6.12 | 3.00 | *[RPS27A, SH3GL2, SHC1]* |
| **R-HSA:2428933** | SHC-related events triggered by IGF1R | 0.002535729 | 22.22 | 2.00 | *[IGF1R, SHC1]* |
| **R-HSA:2428933** | SHC-related events triggered by IGF1R | 0.002535729 | 22.22 | 2.00 | *[IGF1R, SHC1]* |
| **KEGG:04923** | Regulation of lipolysis in adipocytes | 0.002696766 | 5.45 | 3.00 | *[INS, INSR, IRS1]* |
| **R-HSA:2404192** | Signaling by Type 1 Insulin-like Growth Factor 1 Receptor (IGF1R) | 0.002723271 | 5.56 | 3.00 | *[IGF1R, IRS1, SHC1]* |

| | | | | | |
|---|---|---|---|---|---|
| **R-HSA:2404192** | Signaling by Type 1 Insulin-like Growth Factor 1 Receptor (IGF1R) | 0.002723271 | 5.56 | 3.00 | *[IGF1R, IRS1, SHC1]* |
| **R-HSA:2428924** | IGF1R signaling cascade | 0.002736368 | 5.66 | 3.00 | *[IGF1R, IRS1, SHC1]* |
| **R-HSA:2428924** | IGF1R signaling cascade | 0.002736368 | 5.66 | 3.00 | *[IGF1R, IRS1, SHC1]* |
| **R-HSA:1236977** | Endosomal/Vacuolar pathway | 0.002790979 | 18.18 | 2.00 | *[B2M, CTSS]* |
| **KEGG:04216** | Ferroptosis | 0.002937287 | 5.00 | 2.00 | *[FTH1, TFRC]* |
| **R-HSA:5654741** | Signaling by FGFR3 | 0.002937287 | 5.00 | 2.00 | *[RPS27A, SHC1]* |
| **R-HSA:917937** | Iron uptake and transport | 0.002948518 | 5.17 | 3.00 | *[FTH1, RPS27A, TFRC]* |
| **R-HSA:182971** | EGFR downregulation | 0.005312053 | 6.45 | 2.00 | *[RPS27A, SH3GL2]* |
| **R-HSA:2173791** | TGF-beta receptor signaling in EMT (epithelial to mesenchymal transition) | 0.005586252 | 12.50 | 2.00 | *[PRKCZ, RPS27A]* |
| **R-HSA:8875360** | InlB-mediated entry of Listeria monocytogenes into host cell | 0.005586252 | 12.50 | 2.00 | *[RPS27A, SH3GL2]* |
| **R-HSA:76009** | Platelet Aggregation (Plug Formation) | 0.005587752 | 5.13 | 2.00 | *[PTPN1, SHC1]* |
| **R-HSA:9034015** | Signaling by NTRK3 (TRKC) | 0.005796306 | 11.76 | 2.00 | *[IRS1, SHC1]* |
| **R-HSA:9034015** | Signaling by NTRK3 (TRKC) | 0.005796306 | 11.76 | 2.00 | *[IRS1, SHC1]* |
| **R-HSA:1236382** | Constitutive Signaling by Ligand-Responsive EGFR Cancer Variants | 0.006609053 | 10.53 | 2.00 | *[RPS27A, SHC1]* |
| **R-HSA:5637815** | Signaling by Ligand-Responsive EGFR Variants in Cancer | 0.006609053 | 10.53 | 2.00 | *[RPS27A, SHC1]* |
| **R-HSA:2424491** | DAP12 signaling | 0.006633994 | 6.67 | 2.00 | *[B2M, SHC1]* |
| **R-HSA:354192** | Integrin alphaIIb beta3 signaling | 0.006716084 | 7.41 | 2.00 | *[PTPN1, SHC1]* |
| **R-HSA:9006921** | Integrin signaling | 0.006716084 | 7.41 | 2.00 | *[PTPN1, SHC1]* |
| **R-HSA:77387** | Insulin receptor recycling | 0.007471563 | 7.69 | 2.00 | *[INS, INSR]* |
| **R-HSA:8876384** | Listeria monocytogenes entry into host cells | 0.008005403 | 9.09 | 2.00 | *[RPS27A, SH3GL2]* |
| **R-HSA:1643713** | Signaling by EGFR in Cancer | 0.008056271 | 8.00 | 2.00 | *[RPS27A, SHC1]* |
| **R-HSA:982772** | Growth hormone receptor signaling | 0.008481092 | 8.33 | 2.00 | *[IRS1, PTPN1]* |
